# The structural role of osteocalcin in bone biomechanics and its alteration in Type-2 Diabetes

**DOI:** 10.1038/s41598-020-73141-w

**Published:** 2020-10-14

**Authors:** Mahdi Tavakol, Ted J. Vaughan

**Affiliations:** Biomedical Engineering and Biomechanics Research Centre, School of Engineering, College of Science and Engineering, National University of Ireland Galway, Galway, Ireland

**Keywords:** Biomedical engineering, Mechanical engineering, Proteins, Biophysical chemistry

## Abstract

This study presents an investigation into the role of Osteocalcin (OC) on bone biomechanics, with the results demonstrating that the protein’s α-helix structures play a critical role in energy dissipation behavior in healthy conditions. In the first instance, α-helix structures have high affinity with the Hydroxyapatite (HAp) mineral surface and provide favorable conditions for adsorption of OC proteins onto the mineral surface. Using steered molecular dynamics simulation, several key energy dissipation mechanisms associated with α-helix structures were observed, which included stick–slip behavior, a sacrificial bond mechanism and a favorable binding feature provided by the Ca^2+^ motif on the OC protein. In the case of Type-2 Diabetes, this study demonstrated that possible glycation of the OC protein can occur through covalent crosslinking between Arginine and N-terminus regions, causing disruption of α-helices leading to a lower protein affinity to the HAp surface. Furthermore, the loss of α-helix structures allowed protein deformation to occur more easily during pulling and key energy dissipation mechanisms observed in the healthy configuration were no longer present. This study has significant implications for our understanding of bone biomechanics, revealing several novel mechanisms in OC’s involvement in energy dissipation. Furthermore, these mechanisms can be disrupted following the onset of Type-2 Diabetes, implying that glycation of OC could have a substantial contribution to the increased bone fragility observed during this disease state.

## Introduction

Bone is a naturally occurring composite material that consists of hydroxyapatite (HAp) mineral crystals and an organic matrix, which is comprised of both collagenous and non-collagenous proteins. These component phases are hierarchically organised to provide a highly optimised structure that exhibits a multitude of intricate toughening mechanisms that contribute to the tissue’s excellent fracture resistance^1–6^. However, this complex hierarchy presents significant challenges in understanding bone biomechanics and the precise mechanisms by which the component phases dissipate energy during fracture events remains poorly understood. The organic phase of the bone matrix is comprised of approximately 10% non-collagenous proteins^7^. While their contribution towards tissue stiffness and strength is minor^8^, it has become apparent that their role in intrinsic toughening, one of the primary contributors towards healthy tissue’s excellent fracture resistance, is considerable^8–11^. In particular, Osteocalcin (OC) is the most abundant non-collagenous protein in bone^7^. OC is a 49-residue protein that exists as a tertiary structure after carboxylation, with the resulting residues expected to provide a strong binding affinity for the HAp^12^. Together with Osteopontin (OPN), it is responsible for mediating bonding at HAp mineral–mineral interfaces in the extrafibrillar space. Several studies have established that these non-collagenous protein complexes facilitate plastic sacrificial sliding, which appears to be a major contributor to the tissue’s excellent fracture toughness^13–15^. This mechanism manifests at higher length scales in the form of voids that correspond to dilatational bands, which dissipate energy during discrete fracture events^11^ and enable regions of diffuse damage to form under fatigue loading regimes^16^. Crucially, it has been demonstrated through knockout animal models that the capacity of sacrificial sliding is impaired when either OC or OPN are removed, leading to significantly reduced fracture toughness^11,17^ under various loading regimes. While this highlights their critical role in energy dissipation, the underlying molecular mechanisms by which non-collagenous proteins facilitate such behavior remains poorly understood^10,17^. In particular, the strong binding affinity between OC and HAp indicates that adsorption of this non-collagenous protein onto mineral surfaces to ultimately mediate mineral–mineral binding could be a major contributor to bone biomechanics.

Given the important role of non-collagenous proteins in bone energy dissipation, alterations to these proteins has been implicated in certain disease pathologies^8,9^, where bone fragility becomes more prevalent, for instance during Type-2 (T2) Diabetes. T2 diabetes is a multi-factorial disease in which cellular glucose metabolism is disrupted. The resulting hyper-glycemic state allows glucose to form covalent bonds with extracellular matrix proteins throughout the body through a non-enzymatic process known as glycation, resulting in the formation of advanced glycation end products (AGEs)^18–20^.

Protein glycation, or AGE accumulation, disrupts the molecular configuration of extracellular proteins^21^, interfering with their normal biological function. Protein glycation is also thought to play a key role in diabetic bone disease, with studies suggesting that AGE accumulation alters protein mechanics throughout the organic components of the bone matrix, resulting in substantial increases in bone fracture risk among sufferers, up to three-fold in some cases^22,23^. Until now, the vast majority of studies exploring AGE accumulation have focused on glycation of the collagen network^24–27^, with studies clearly identifying candidate cross-link locations^27^ and using molecular dynamics simulation to identify that increased cross-linking ultimately stiffens the collagen network^28^. In this context, collagen has been vastly explored under the premise that only proteins having long half-life undergo glycation, leaving the short-lived NCPs understudied^29^. However, there is evidence for glycation of short-lived proteins in the literature^30^ and given the important role that NCPs have in energy dissipation, their glycation could have a detrimental role in overall bone biomechanics. As yet, the effect of glycation on the mechanical role of OC has not been investigated.

Although there are limited evidence of OC glycation in the literature^31,32^, a recent experimental study has demonstrated that OC can become glycated through a post-translational modification at the N-terminus, which could have important implications on energy dissipation potential of this NCP. While this study suggests that this glycation was unlikely to interfere with OC's interaction with HAp, it is notable that these findings were based on a fragmented OC protein in which the fragmentation occurred between 19 and 20 residues. In the case of the full 49-residue polypeptide, it is possible that other candidate glycation sites exist and that glycation could lead to structural changes in the protein structure, ultimately affecting its intended role^33^. Given the critical toughening role that OC has at mineral–mineral interfaces in the tissue, any structural alterations brought about by T2 diabetes could have severe implications for binding affinity and OC’s capacity to dissipate energy during bone fracture events.

In this paper, a molecular dynamics (MD) framework is used to investigate OC mediated energy dissipation on a HAp mineral substrate through a systematic evaluation of (i) surface adsorption and (ii) subsequent pulling simulations. This study considers the full 49-residue polypeptide representation of OC and uses this to explore the effect of glycation on the overall energy dissipation potential, providing novel insight into how OC protein mechanics are altered during T2 Diabetes.

## Methods

### Background

OC mediated energy dissipation was investigated through a MD framework^34^ that considers interactions between the full 49-residue polypeptide and a HAp mineral crystal. In the first instance, adsorption of OC onto the HAp surface was simulated to obtain the protein arrangement on the mineral surface. Following this, a Steered Molecular Dynamics (SMD) model was used to simulate OC protein pulling from mineral surface to evaluate energy dissipation. The study also investigates the role of glycation on the OC structure to determine its potential effect on adsorption and subsequent energy dissipation.

#### HAp structure

HAp is the main mineral phase of the bone and has an ionic structure with the chemical formula of ${\text{Ca}}_{10} \left( {{\text{PO}}_{4} } \right)_{6} \left( {{\text{OH}}} \right)_{2} .\user2{ }$ Due to the symmetries present in its crystal structure, it can be considered as the space group ${\text{P}}6_{3} /{\text{m}}$^35^ that has unit cell parameters of 9.41 Å, 6.88 Å, and 2 Å. In these simulations, the mineral thickness is 38.0 Å and OC proteins are considered to interact with the main (010) facet of the crystal which contributes to ~ 70% of HAp mineral surfaces in bone^36^.

#### Healthy OC protein structure

**Figure 1:**
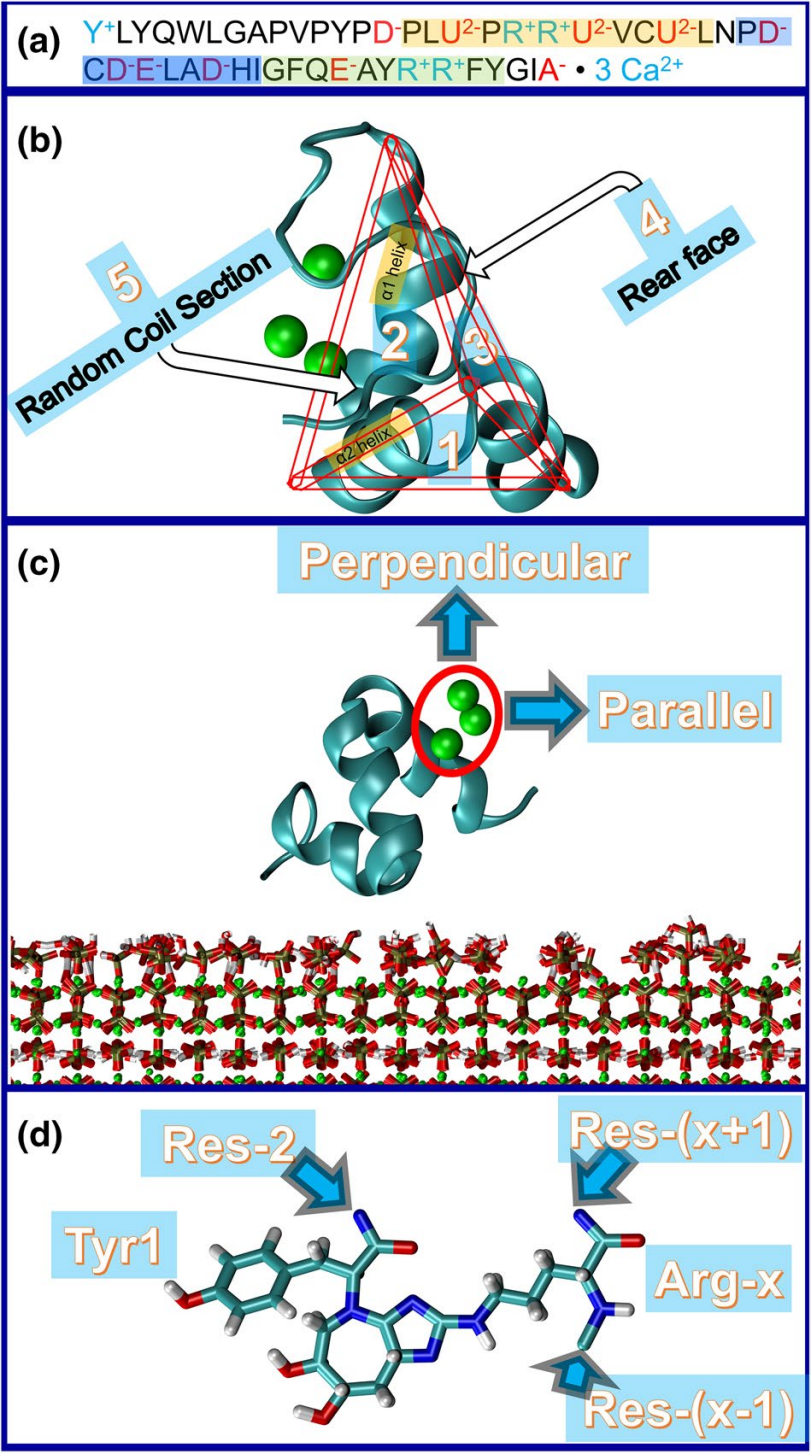
Simulation setup: (**a**) The amino-acids sequence and the secondary structure content of the protein. Residues are colored and highlighted based on their electrical charge and their helix. The residues without any secondary structure content have not been highlighted and the protein has three surface bound calcium ions. (**b**) Five different initial orientations considered in the adsorption simulations of healthy OC. (**c**) The SMD pulling simulations were done in two different orientations of parallel and perpendicular. (**d**) The structure of the cross-link considered in the current study. The main residues are TYR1 and ARG43 and the neighbouring residues of the cross-link are shown with arrows.

While OC is a 49-residue protein (see Fig. 1a), previous molecular dynamics simulations have generally considered amino-acids 13–49 based on the structure (code 1Q8H) available in the Protein Data Bank (PDB)^37,38^. This study considers the full OC protein by using the Molefacture plugin of the Visual Molecular Dynamics (VMD) package^39^ to build the remaining portion of the protein which exists as a random coil segment^40^. 15 simulations with different initial random seed numbers having a total simulation time of 180 ns were started to generate a complete OC structure. Even though the final structures of the random coil segment varied, their potential energies were in the same range. Starting from any of these initial structures, it is reasonable to assume that a very long simulation framework will sample many of these configurations. As a result, one of these structures was chosen as a representative for the whole protein (see Fig. 1b).

#### Glycated OC protein structure

The most common AGE cross-link, glucosepane, forms between arginine and lysine residues^27^. However, OC does not contain any lysine residues. Moreover, in an experimental study by Thomas et al. the formation of glycation adducts in the N-terminus was discovered^32^. Considering that glucosepane has a 1000 times higher abundancy than the other AGEs^27^, the authors beleive that it is reasonable to consider that a cross-link similar to glucosepane was formed (see Fig. 1d). Besides, the glycation adducts are highly reactive and they can react with amino, guanidino and sulfhydryl groups^33^. As the cysteine residues of Osteocalcin take part in a disulphide bond^12^, their sulfhydryl groups are not available for AGE formation. The guanidino groups of the arginine residues are the only candidates for AGE formation since the protein does not have any lysine residues.

Thus, the formation of a glucosepane like cross link between arginine and N-terminus is considered here. As well, in the 15 simulations carried out for healthy complete OC equilibration (section Healthy OC Protein Structure), there is no evidence for the N-terminus proximity to ARG19, ARG20 or ARG44 in contrary to ARG43. As a result, the amino group of the N-terminus and side chain of ARG43 are chosen for cross-link formation. To this end, three different simulations of soluble complete OC were carried out, whereby a hypothetical bond was first created between N-terminus and ARG43 to bring them close together for the formation of an AGE crosslink. Finally, the ARG and N-terminus came into close vicinity of each other. Then, a glucosepane crosslink between them was formed by removing extra hydrogen atoms and adding new bond, angles and dihedrals to the system.

### Model cases

#### Adsorption

Adsorption for the healthy case was considered along the five main faces of the protein structure (Fig. 1b). During the generation of the random coil segment it was observed that this segment did not configure itself on the faces 1–4. Therefore, the reduced structure (amino acids 13–49) was used to examine orientations 1–4. Meanwhile, the full protein structure, complete with the random coil segment, was used to explore the 5th orientation. To explore the role of the random coil in detail, this case itself was subdivided into four different orientations. Three different simulations with the initial minimum protein surface distances of 5 Å, 4 Å and 3 Å were initiated for each arrangement. Each simulation is named based on its orientation, the initial distance and its simulation number. For instance, R1-4A-#2 refers to the second simulation in which the protein is placed in the first orientation with 4 Å minimum distance. In the adsorption simulation, residues having at least one heavy atom in the 4 Å vicinity of the HAp surface were considered as the contact residues. A code was written to enumerate the number of frames in which each residue has been among the contact residues. For the glycated case, the protein secondary structure was actually disrupted and therefore the same protein facets were not identifiable. For this glycated case, six different orientations were examined using the same simulation protocol to that described above.

#### Energy dissipation

In adsorption simulations, the potential energy was averaged in 1 ns timeframes. Out of many simulations done for each case, those with the lowest final potential energy were chosen to study the energy dissipation, unless stated otherwise. SMD simulation was utilized to investigate the relative movement of OC with respect to the HAp surface in the extrafibrillar matrix^41^ by pulling in both parallel and perpendicular directions with respect to the HAp surface (Fig. 1c). As the protein does not adsorb on the HAp surface from its three surface bound Ca^2+^ ions in almost all the simulations, these ions were tagged as the SMD atoms to pull the protein^42^. The SMD spring constant was initially chosen as 10 kcal/(mol Å^2^) to represent the stiffness of Osteopontin, which is usually found in a complex with OC at mineral interfaces. However, this stiffness may vary due to the Osteopontin random coil structure^11^ and a parameter study around spring constant is presented for one of the cases. Resulting force–displacement curves were used to calculate the dissipated energy during the SMD simulation, defined as the cumulative work (e.g. the sum of the work done during short time periods). Details related to the choice of the pulling speed and the verification of SMD method are presented in the SI**.**

### Model parameters

The initial model and forcefield parameters for HAp were taken from the CHARMM-Interface forcefield^36^. The PDB code of 1Q8H has three unnatural amino acids called γ-Carboxy Glutamate (or GLA) keeping the surface bound Ca^2+^ ions^43,44^. The forcefield parameters for this residue were taken from a previous study by Tavakol et al.^42^. Energy minimization and equilibration were done for the duration of 50,000 steps and 100 ps before the adsorption simulation. During these stages, the carbon backbone atoms and the lower layer of HAp having a thickness of 2 Å were constrained. The constraint on the backbone atoms was removed for the adsorption simulations. In all the simulations, temperature and pressure were kept constant at physiologically relevant values of 310 K and 1 bar, respectively. To neutralize the simulation box and have a correct electrolyte concentration, Na^+^ and Cl^−^ ions with the total concentration of 150 mM were added to the system. The rest of the simulation details are mentioned in a previous study^42^.

## Results and discussion

### Healthy OC

#### Adsorption

**Figure 2:**
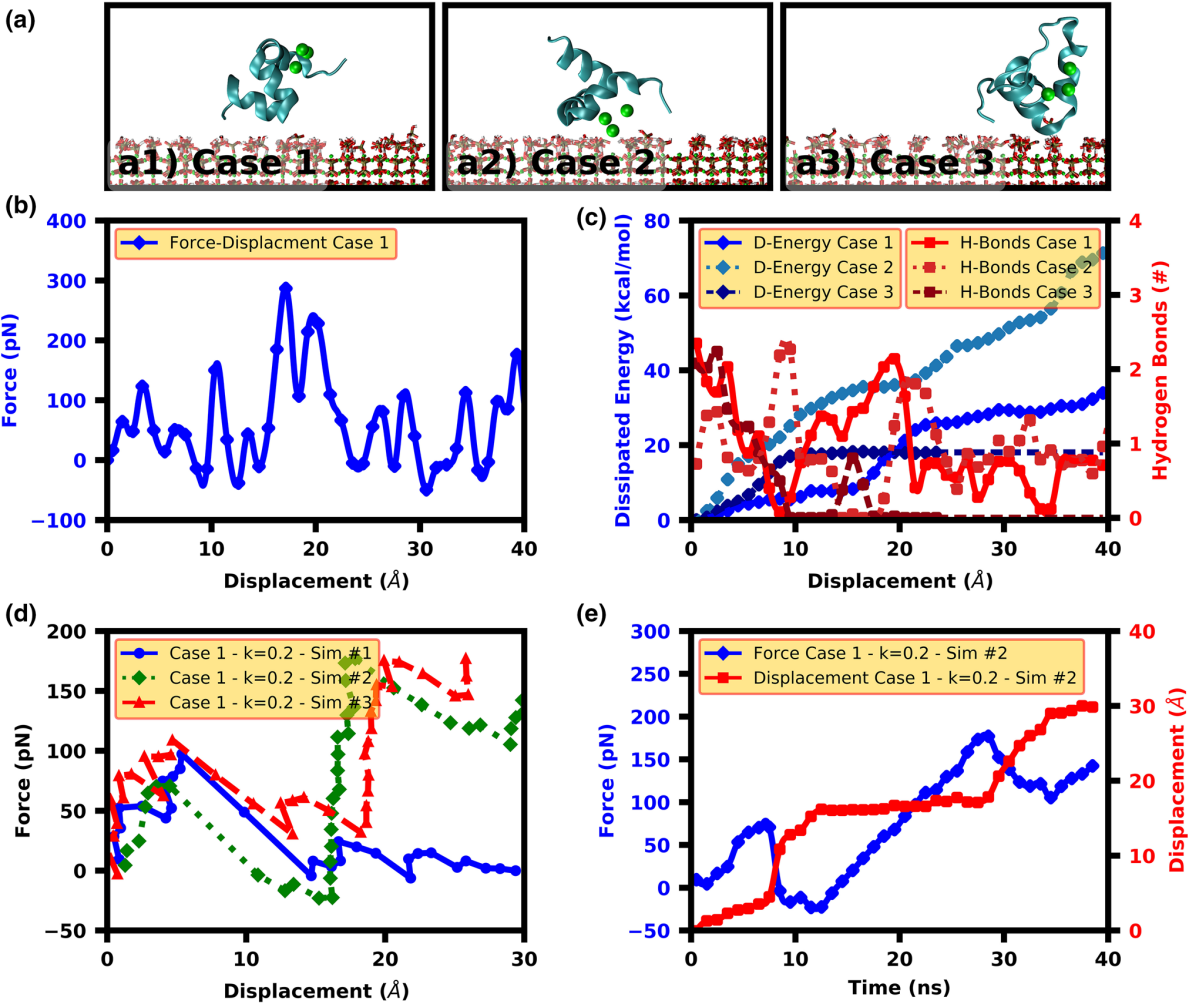
SMD results for OC parallel pulling. (**a**) Initial configurations used in SMD simulation. (**b**) An instance of force–displacement curve (case-1) utilized in calculating the dissipated energy. (**c**) The dissipated energy and hydrogen bond curves for three different cases shown in (**a**) illustrates a direct relation between hydrogen bonds and energy dissipation rate. These curves also show higher energy dissipation for the case-2 where the protein adsorption happens from its calcium ions. (**d**) Force–displacement curves for the case-1 with lower spring constant and (**e**) Force and displacement–time curves for the simulation #2 (panel d) illustrate the stick–slip mechanism.

The contact residues for the adsorption of the healthy OC on HAp for different initial orientations and protein surface distances are provided in Table 1 (For the complete table see Table SI1). For each orientation, parts of the protein that face toward the surface at the beginning of the simulation are highlighted in yellow, while X denotes the contact residue. For each α-helix, there were exactly six simulations where the helix faced toward the surface at the beginning of the simulation that resulted in adsorption. While this indicates that there is no orientation-related bias toward any α-helix, the results show that there were more instances of protein adsorption from the α2-helix than any other helix. The underlying reason is that the α2-helix has four negatively charged residues dispersed in the amino acid sequence that have an opportunity to interact with ${\text{Ca}}^{2 + }$, ${\text{HPO}}_{4}^{2 - }$ and ${\text{H}}_{2} {\text{PO}}_{4}^{ - }$ ions on the HAp surface. Contrary to this, the α3-helix has two Arginine residues located beside each other that are positively charged, while the α1-helix has γ-Carboxy Glutamate residues whose negative charges are canceled by the protein surface bound ${\text{Ca}}^{2 + }$ ions, with the arginine residues are protected by this motif of the protein. The main contact residues are HIS35, GLU31, ARG44, ARG43 and ASP28 with the percentages of 24.43%, 23.99%, 19.41%, 18.85% and 16.13%, respectively. Simulation R1-4A-#2 (see “Methods/HAp Structure” section for naming convention), which was adsorbed through the α2-helix, was chosen as Case 1 for SMD simulation as it had the lowest potential energy (Fig. 2a1). Meanwhile, simulation R3-4A-#2 was also chosen for SMD as Case 2, as it adsorbed on the surface on the α3 helix through the 3 ${\text{Ca}}^{2 + }$ protein motif (Fig. 2a2), which presented an interesting binding feature that warranted further investigation. For the fifth orientation, the most important contact residues are TYR1, ASP28, PRO27, GLU31 and ARG44 with the percentage of 37.74%, 28.82%, 24.24%, 19.86% and 16.18% among the contact residues, respectively. Finally, Simulation R54-3A-#1 was also chosen as it showed adsorption through the random coil section. More details about the simulation results for the adsorption of healthy OC in the fifth orientation are summarized in the SI.

**Table 1:**
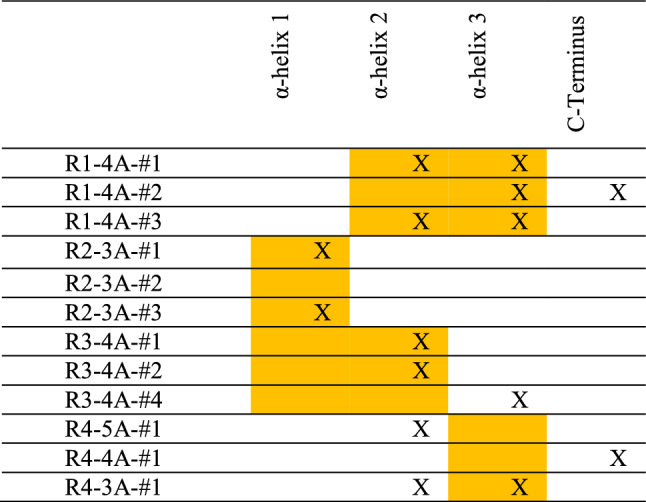
The contact helices of the adsorption of healthy OC on HAp.

The contact residues are shown with “X”, while those parts of the protein that face toward the surface at the beginning of the simulation are highlighted in yellow.

#### Energy dissipation mechanisms for parallel pulling

The force–displacement behavior at a pulling speed of 1 Å/ns was used to calculate energy dissipation of each configuration (see Fig. 2a,b). A value of $30.01 \pm 5.23{ }\;{\text{kcal}}/{\text{mol}}$ was calculated as the total energy dissipation for the Case 1 (α2-helix binding). The energy dissipation curve (Fig. 2c) highlighted the presence of a *stick*–*slip* mechanism under this deformation mode, where increases in dissipation energy coincided with changes in the number of hydrogen bonds. The dissipated energy curve (Fig. 2c) shows several time intervals of zero energy dissipation (horizontal segments) and several other intervals of high energy loss. This was also seen in other simulations with different random seed number or pulling speed of 10 Å/ns (Fig. SI1). To provide further insight into this mechanism of energy dissipation, a lower SMD spring constant of 0.2 kcal/mol Å^2^ was chosen to slow the *stick–slip* process, making it more identifiable. Here, the force–displacement curves (Fig. 2d) illustrate tangible drops and jumps in pulling force reminiscent of the stick–slip mechanism. The force–time and displacement–time curves for one of the simulations (Fig. 2e) shows that during the time interval of 0–8 ns, the displacement does not change and the force increases at the same time (the stick stage). At 8–12 ns time interval, there is a jump in the displacement that coincides with a distinct drop in the pulling force (e.g. slip stage). Then, the displacement remains the same and the force increases (the 2nd stick) until 29 ns when an increase in the displacement causes a drop in the force value (the 2nd slip). Afterwards, there are final stick and slip stages and the protein remains adsorbed on the surface at the end of the simulation with the possibility of having more stick–slip cycles. The HAp charge periodicity in the pulling direction for the parallel pulling is one of the underlying reasons for this stick–slip mechanism and similar observations were made across all three simulation cases with different random seed numbers (Fig. 2d).

It is notable that the total energy dissipation for Case 2 (3 ${\text{Ca}}^{2 + }$ binding) was $56.26 \pm 7.53\;{\text{ kcal}}/{\text{mol}}$, approximately twice the corresponding value measured for Case 1 (α2-helix binding) (Fig. 2a). Here, energy dissipation becomes further enhanced by a unique mechanism observed through the 3 ${\text{Ca}}^{2 + }$ motif becoming adsorbed on the HAp surface a few nanoseconds after pulling initiates (Fig. 2a2). These contact residues meant that OC never desorbed from the HAp surface in this configuration, which resulted much higher levels of energy dissipation compared to the previous case. This unique role of the 3 ${\text{Ca}}^{2 + }$ motif in the energy dissipation has not previously been reported and, together with the stick–slip mechanism, may contribute to the excellent energy dissipation provided by OC at these interfaces.

In Case 3, where the random coil adsorbed onto the surface along with portions of the α2-helix (Fig. 2a3), there is a reduced energy dissipation capacity, arising from a lower number of stick–slip cycles (Fig. SI2), compared to the cases where the α-helices directly face toward the HAp surface (Fig. 2c). In this orientation, the contact residues are TYR1 (N-terminus) from the random coil segment and ASP28 and GLU31 from the parts of the α2-helix. In this simulation, the parallel speeds of $0.08 \pm 0.22{ }\;$ Å/ns, $0.53 \pm 0.04\;$ Å/ns and $0.15 \pm 0.08{ }\;$ Å/ns for TYR1, GLU31 and ASP28 are lower than the pulling speed of 1 Å/ns for the spring connected to the 3 ${\text{Ca}}^{2 + }$ motif of the OC. The lower speeds of the contact residues show the stick stage of the stick–slip mechanism. Furthermore, the lower speed of TYR1 and ASP28 compared to than GLU31 indicates that the GLU residue takes the majority of deformation before desorption, pointing to the presence of a sacrificial bond mechanism between the two binding points on OC and the HAp surface.

#### Energy dissipation for perpendicular pulling

In pulling the protein perpendicular to the HAp surface, the maximum force reaches a plateau for pulling velocities of lower than 1 Å/ns (Fig. SI3a), with maximum forces across all configurations lower than those observed for parallel pulling (Fig. 2c). Importantly, the simulations with different random seed numbers peak at the similar positions for perpendicular pulling in contrast to the parallel case. At the force–displacement curves for the same simulations with pulling distance of 9 Å, the protein desorbs from the surface and the dissipated energy in this case is equal to $7.31 \pm 1.40\;{\text{ kcal}}/{\text{mol}}$ (Fig. SI3b). Also, like the parallel pulling simulation there is a direct relation between the number of hydrogen bonds and the energy dissipation rate (Fig. SI3b). However, the stick–slip process is absent in the perpendicular orientation, which results in lower energy dissipation in this configuration.

### Glycated OC

#### Adsorption

**Figure 3:**
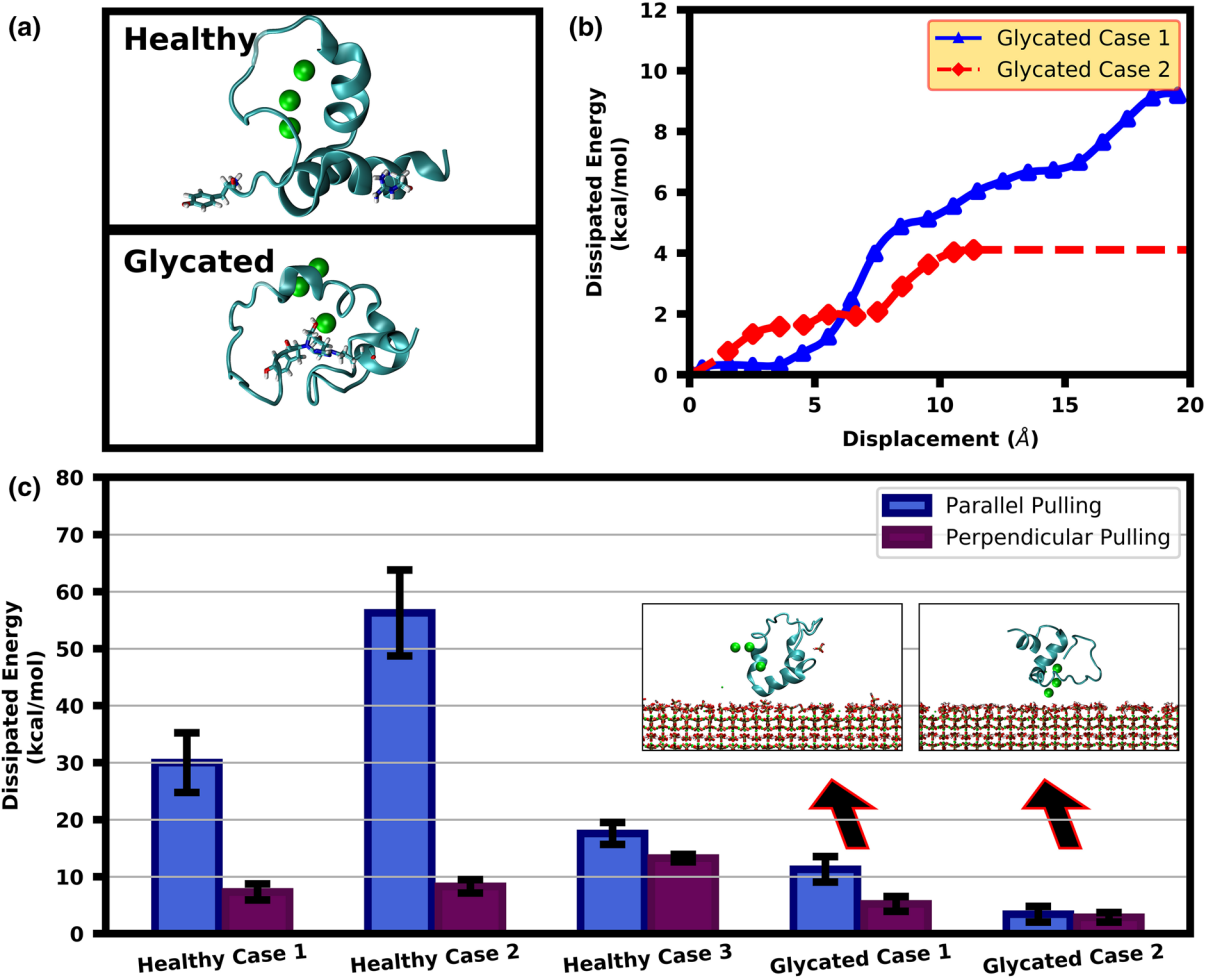
T2-Diabetes effect on OC energy dissipation. (**a**) The formation of AGE crosslinks in diabetes OC leads to the disruption of secondary structure. The aminoacids related to AGE formation are shown with different representation. (**b**) Energy dissipation curves for parallel pulling of diabetes OC illustrates different energy dissipation for different protein configurations on the surface. (**c**) The parallel and perpendicular pulling energy dissipations amounts for healthy and diabetes OC show that during the diabetes OC loses both of its energy dissipation mechanism of calcium ion mediated and stick–slip because of its lower helix content.

Exploring the effect of T2 Diabetes on OC, it was found that glycation of OC could possibly disrupt the protein’s α-helix structures as shown in Fig. 3a. Based on six different orientations and three separate initial distances, it was found that the glycated OC protein had less affinity to the HAp crystal in the absence of some α-helice secondary structure contents, with protein adsorption happening in only 2 out of 18 simulations. The main contact residues for these two cases are PRO27, GLA21, LEU25, GLU31 and ASP28 having the percentages of 39.35%, 38.25%, 33.90%, 31.00%, 28.90% among the contact residues, respectively. Comparing the glycated OC case with the healthy OC structure, the absence of residues TYR1 and ARG43 is evident as the formation of AGE crosslink between them resulted in elimination of their electrical charge.

#### Energy dissipation

SMD pulling simulations were carried out for the two glycated OC cases where protein adsorption occurred (Fig. 3b), with both cases showing reduced energy dissipation capacity compared to all the healthy OC cases. For Case 1, the glycated OC protein adsorbs onto the HAp surface from its ASP28, GLU31 and HSD35 residues and shows limited evidence of stick–slip instances or sacrificial bonds (Fig. 3), with a total energy dissipation value of $11.28 \pm 2.23{ }\;{\text{kcal}}/{\text{mol}}$ observed. For Case 2, the glycated OC protein adsorbs onto the HAp surface from its 3 surface bound ${\text{Ca}}^{2 + }$ ions (Fig. 3c). The energy dissipation of this case is lower than the corresponding healthy OC where the surface bound ${\text{Ca}}^{2 + }$ ions face toward the HAp surface (e.g. Healthy Case 2 in Fig. 2). The beneficial dissipation mechanism of the 3 ${\text{Ca}}^{2 + }$ motif identified in the healthy case does not manifest in the glycated case, with a lower energy dissipation of $3.41 \pm 1.38\;{\text{ kcal}}/{\text{mol}}$ reported.

The energy dissipation for perpendicular pulling of glycated case 1 and 2 are $5.22{ } \pm 1.30{ }\;{\text{kcal}}/{\text{mol}}$ (Fig. 3c) and $2.87{ } \pm 0.84\;{\text{kcal}}/{\text{mol}}$, respectively. Considering the error bars, the values for the healthy and glycated OC proteins are very close to each other. In the perpendicular pulling the number of contact residues are limited and after a contact residue is desorbed, other residues cannot take part in the adsorption because of their large distances from the HAp surface. Thus, there is neither a stick–slip mechanism nor any effect from the protein structure, resulting in similar dissipated energies of healthy and glycated OC under perpendicular deformation.

### α-Helices importance in energy dissipation

The results here demonstrate that α-helix structures in the OC protein play a critical role in energy dissipation behavior. In the first instance, α-helix structures have high affinity with the HAp surface and provide the most favorable conditions for adsorption of OC under normal conditions. In pulling simulations, several key energy dissipation mechanisms directly associated with binding at α-helix structures were observed that included stick–slip behaviour, a sacrificial bond mechanism and favorable binding feature provided by the 3 ${\text{Ca}}^{2 + }$ motif on the OC protein.

The stick–slip mechanism is present through the α-helix because there is a periodicity in the electrical charges on the HAp surface. Each contact residue avoids the movement parallel to the surface because of its favorable electrical interactions with the HAp ions they are in contact with and having unfavorable affinity toward the neighboring ions. The rigidity of the helix does not let the residues avoid the unfavorable adsorption modes through protein deformation during the protein movement. Thus, they are stuck on the surface unless the force is high enough to break their favorable interactions with the HAp contact ions and then the contact residues slide on the surface to the next favorable position in the parallel orientation. This mechanism was further enhanced by two-point sacrificial bonding mechanism observed between TYR1 and the HAp surface. It has previously been hypothesized that sacrificial bonds play a major role in energy dissipation in non-collagenous proteins at HAp interfaces, and is a major contributor to bone fracture resistance^10^, with these results here providing the first molecular-level evidence of this mechanism being involved. It is worth noting that in all cases, energy dissipation in OC is independent of backbone stretching, with no evidence of energy being dissipated through unfolding of the protein (commonly hypothesized to dissipate energy in other proteins^45^). Here, the main mechanisms of energy dissipation are in-fact dependent on the rigidity of the protein and are mediated through non-covalent interactions at α-helix binding interfaces.

In the case of Type-2 Diabetes, the protein becomes glycated through covalent crosslinking between Arginine and N-terminus regions, which causes disruption of most of its α-helix content. Interestingly, the loss of secondary structures upon protein glycation has been observed previously in hemoglobin^21^, where an increase in beta-sheet and random coil secondary structure elements at the expense of α-helices was observed, similar to the structural changes observed for OC in the current study. The disruption of these secondary structures has significant implications for OC’s capacity of energy dissipation. Glycated OC has lower affinity to the HAp surface and this disrupted configuration loses much of its energy dissipation capacity, as the energy dissipation mechanisms are impaired with the presence of less α-helix structures in OC. For the glycated cases, the beneficial effect of the 3 ${\text{Ca}}^{2 + }$ motif is not observed in glycated OC, because of the ${\text{Ca}}^{2 + }$ ions desorption from the surface. The underlying reason behind this behavior is because the lack of a secondary structure in the protein around this motif (Fig. 3a) implies that protein deformation is easier, which leads to desorption of the ${\text{Ca}}^{2 + }$ ions from the surface. Furthermore, in the absence of α-helix structures, sacrificial bond or stick–slip mechanisms were no longer observed. For the latter, protein segments without a secondary structure can avoid unfavorable contact during protein movement parallel to the HAp surface through protein backbone deformation as there are fewer hydrogen bonds in the backbone than in helix or beta sheet segments. Thus, there is no need to have a stick stage to prevent unfavorable contacts since there are less hydrogen bonds to avoid the backbone deformation leading to a more continuous protein movement. Together, these observations highlight the critical role that α-helices have in energy dissipation at HAp interfaces.

## Conclusions

This study provides novel insight into the molecular mechanisms responsible for energy dissipation of OC at HAp mineral interfaces in bone tissue. In particular, the results demonstrate that the protein’s α-helix structures play a critical role in energy dissipation behavior, having high affinity with the HAp surface and enabling several critical energy dissipation mechanisms during pulling. In the case of Type-2 Diabetes, this study demonstrated that glycation of the OC protein caused disruption of α-helices structures, resulted in significantly impaired energy dissipation capacity due to i) lower protein affinity towards the HAp surface and ii) reduced capacity of glycated OC to dissipate energy. This study has significant implications for our understanding of bone biomechanics in Type-2 Diabetes, revealing a potential role for glycation of the non-collagenous component of the organic matrix in increased bone fragility during the disease.

## Supplementary information

Supplementary Information 1.

Supplementary Information 2.

## Data Availability

All the files necessary to reproduce the results reported here alongside with the processed simulation data are openly available in Figshare at “https://figshare.com/s/3a3fe8559501221e69c1”.
