# Supplementary material for: The structural role of osteocalcin in bone biomechanics and its alteration in Type-2 Diabetes
Source: Sci Rep. 2020 Oct 14;10:17321. doi: 10.1038/s41598-020-73141-w (PMC7560881; doi:10.1038/s41598-020-73141-w)
Supplement: Supplementary file 1 — Supplementary Information 1. [file 41598_2020_73141_MOESM1_ESM.zip › SI/SI_v19.pdf]

Type-2 Diabetes Effect on the Role of Osteocalcin in Bone Biomechanics

Mahdi Tavakol, Ted J. Vaughan*

Biomedical Engineering and Biomechanics Research Centre, School of Engineering, College of Science and Engineering, National University of Ireland Galway, Galway, Ireland.

Address for correspondence:

Dr. Ted J Vaughan
Senior Lecturer in Biomedical Engineering,
Biomechanics Research Centre (BMEC)
Biomedical Engineering
National University of Ireland Galway
Galway
Ireland
Phone: (353) 91-493084
Email: ted.vaughan@nuigalway.ie

Keywords
Bone Biomechanics, Type-2 Diabetes, Osteocalcin, Advanced Glycation Endproducts

Supplementary Figures

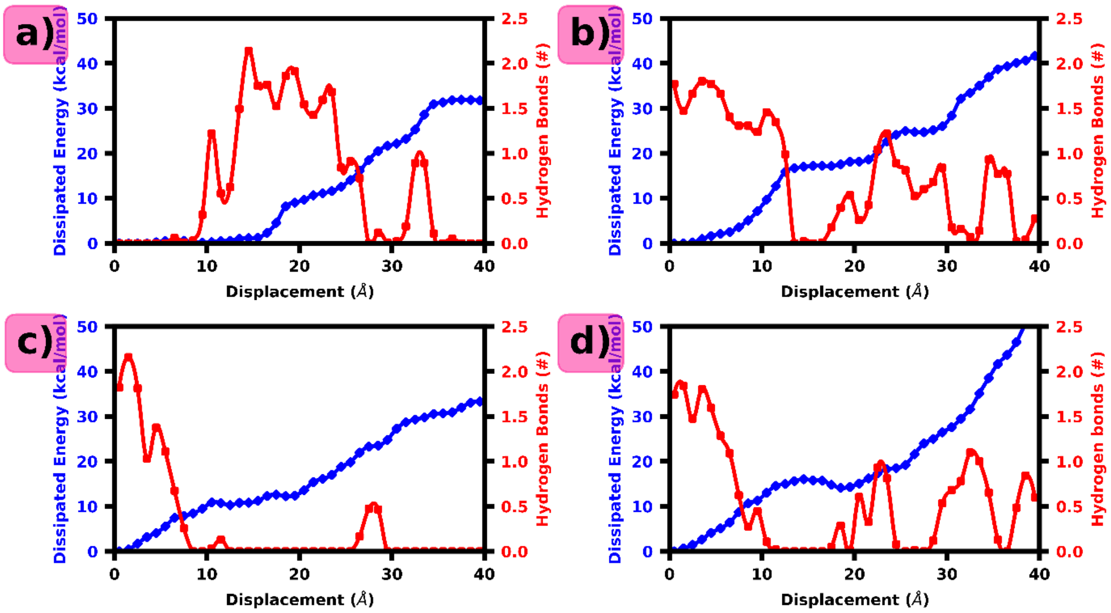

Figure SI1. The dissipated energy and number of hydrogen bonds. These curves show a direct relation between the energy dissipation rate and the number of hydrogen bonds for different simulations of R1-4A-#2 for parallel pulling.
a) 1 Å/ns simulation #4, b) 1 Å/ns simulation #5, c) 10 Å/ns simulation #1 and d) 10 Å/ns simulation #3.

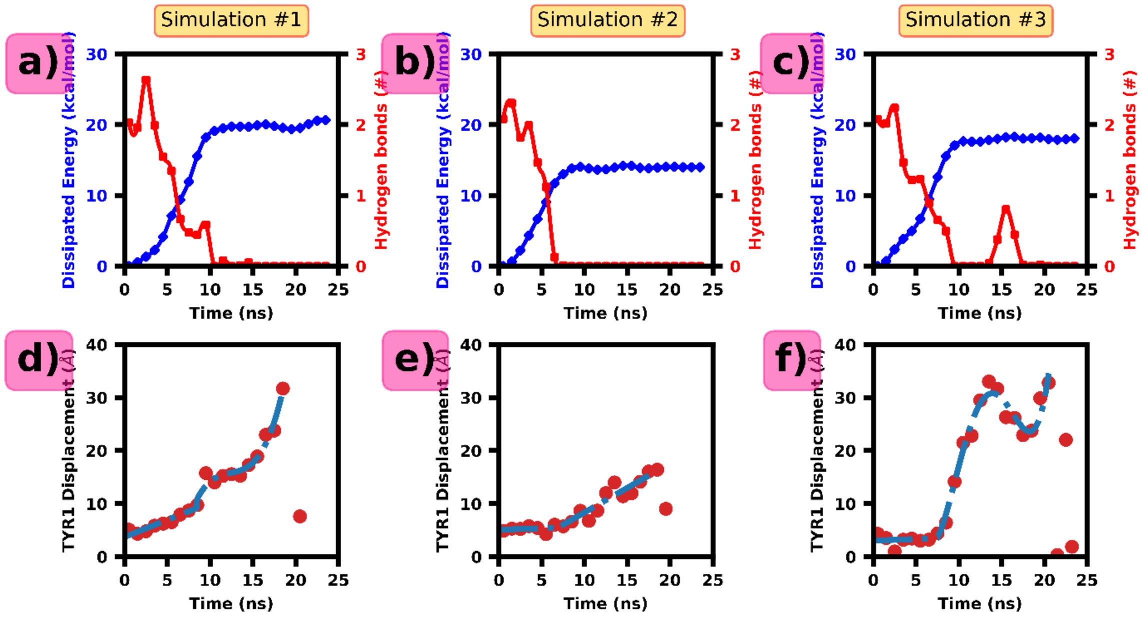

Figure SI2. The simulation results for the parallel pulling of OC in healthy Case 3 (R54-3A-#1). These results show the stick-slip mechanism occurrence. Dissipated energy and number of hydrogen bonds for a) simulation #1, b) simulation #2 and c) simulation #3. TYR1 displacement for d) simulation #1, e) simulation #2 and d) simulation #3. As long as the TYR1 is stuck on the surface (or its parallel pulling velocity is lower than the pulling velocity) the dissipated energy increases. After a jump in the TYR1 displacement there is no more energy dissipation (fixed total dissipation amount. e.g. horizontal curve)

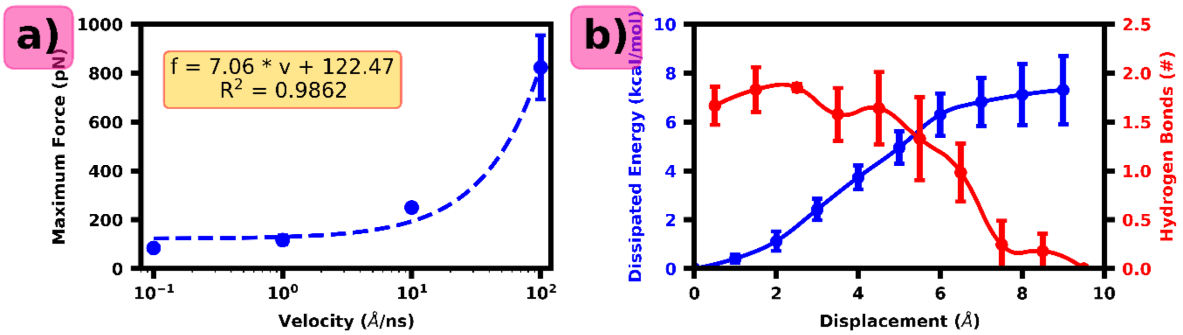

Figure SI3. The simulation results of the SMD simulation of R1-4A-#2 for perpendicular pulling. a) the maximum force – velocity shows the maximum force convergence for the pulling speeds of lower than 1 Å/ns. b) Dissipated energy (blue curve) and hydrogen bond number (red curve) – displacement curves for the pulling speed of 1 Å/ns averaged over three simulations with different random seed numbers illustrating a direct relation between the hydrogen bonds number and the energy dissipation rate.

Method

Model Cases

Energy Dissipation

Final snapshot of the simulation R1-4A-#2 (Table SI1) is utilized to run SMD simulations with pulling velocities between $1 - 100\,\text{Å}/ns$ to obtain a competent velocity value. The maximum force – velocity curve reaches a plateau before the velocity of $10\,\text{Å}/ns$ (Figure SI4-a), with simulations carried out at the lowest velocities of $1\,\text{Å}/ns$ and $10\,\text{Å}/ns$ showing similar behavior, even though each simulation peaks at different displacement value (Figure SI4-b). This behavior can be considered as a reminiscent for a mechanism with a non-deterministic nature.

In physiologically relevant pulling velocities, the covalent bonds in the OC protein do not bear external loading. Instead, load is transmitted through non-bonded interactions. For the pulling speed of $1\,\text{Å}/ns$ the maximum force is equal to $256.09 \pm 28.31\; pN$. If the whole protein backbone is under this force, according to Thompson et al, the carbon-carbon bond energy will be equal to $0.7\; kcal/mol$ which is a little higher than a kT and is around 2 percent of the dissipated energy[1]. In addition, the strength of Ca^{2+} mediated ion networks in osteopontin which are also present in the OC-HAp interface is estimated to be between $100\; pN$ and $1100\; pN$[2]. The value of $256.09 \pm 28.31\; pN$ measured here is in this range. Thus, the selected pulling rate is small enough for the purpose of the current study.

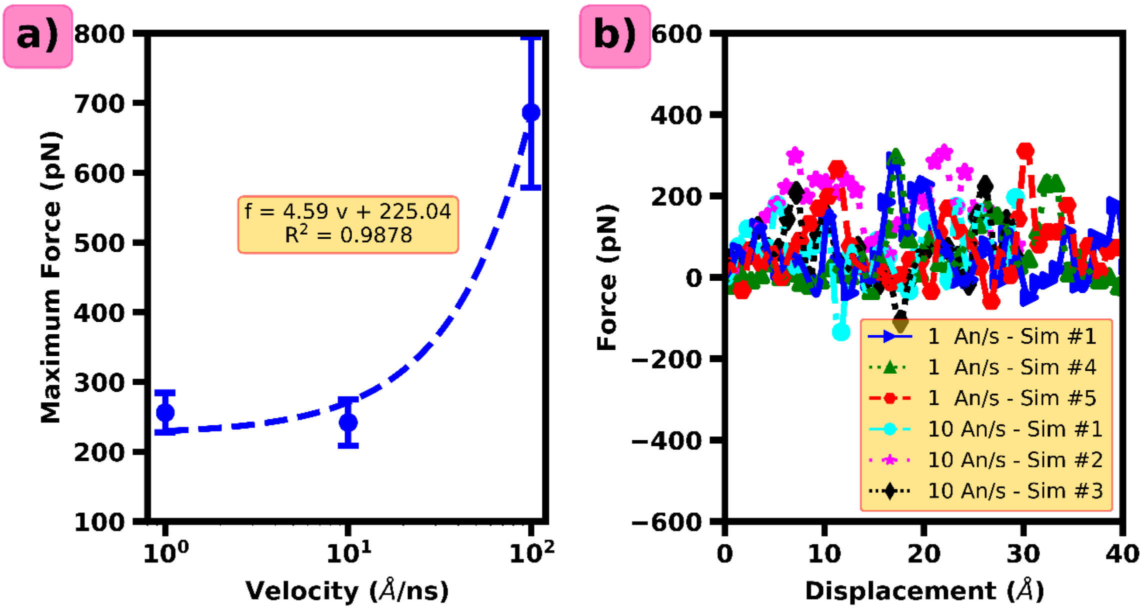

Figure SI2. The effect of pulling velocity on the SMD simulation of R1-4A-#2 for parallel direction. a) The maximum force – pulling velocity curve shows the converge in the maximum force after reaching the speed of 1 Å/ns. b) The force-displacement for simulations done with pulling speeds of 1 Å/ns and 10 Å/ns using different random seed numbers shows the presence of a non-deterministic mechanism.

Results and Discussion

Healthy OC

Adsorption for 5th Orientation

As mentioned before, the fifth configuration was itself subdivided into four initial orientations. For this case just the orientations in which the random coil segment (residues 1-12) faces toward the HAp surface are considered. For this case the most important contact residues are TYR1, ASP28, PRO27, GLU31 and ARG44 with the percentage of 37.74%, 28.82%, 24.24%, 19.86% and 16.18% among the contact residues, respectively. The final simulation snapshots for R51-3A-#2 and R54-3A-#1 are almost the same as each other (Figure SI5). The protein adsorption results showed that for the simulation R51-3A-#2 the protein rotates and switches to an orientation like the simulation R54-3A-#1. As a result, it seems that the former had an opportunity to opt to other orientations and it chooses the latter because of its favorability. Thus, the final snapshot of R51-3A-#2 is selected for the SMD simulation and surprisingly enough its main contact residues are TYR1, PRO27, ASP28 and GLU31 similar to the most prominent contact residues among all the simulations for the complete OC adsorption on HA.

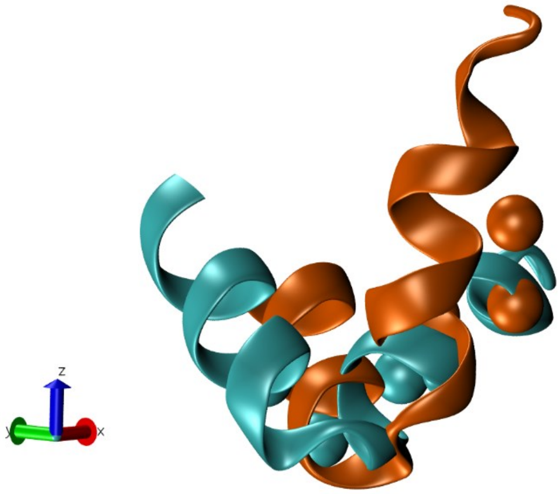

Figure SI3. The final orientation for the adsorption of the complete OC. The final configuration for the simulations R51-3A-#2 and R54-3A-#1 are the same. In this figure just the 2nd and 3rd helices of the protein are shown and the R51-3A-#2 and R54-3A-#1 are coloured in bronze and blue respectively.

Energy Dissipation for Parallel pulling

The contact residues during the SMD pulling for three simulations done at $1\,Å/ns$ are summarized in Table SI2. At the beginning of the simulation, the main contact residues are GLU40 and ARG43 like table SI1. As the simulation proceeds, the helix in contact with the HAp surface switches from α3 to α2. After this change, the contact residues of protein are ASP28, GLU31, ASP34 and HSD35. It seems that the protein rolls over on the surface during the pulling and it finds itself a preferable contact mode with the HAp surface. It is interesting that the main contact part of the protein switches to α2 helix which showed more importance in the adsorption simulations (Table SI1).

To shed more light on the OC mediated energy dissipation, SMD simulations with lower spring constants of 2 and 0.2 $kCal/mol/Å^2$ were carried out as lower SMD spring constant slows down the process. Changes in the spring constant does not affect the amount of dissipated energy for the higher spring constants (Figure SI6). For the lower spring constant of $0.2\,kCal/mol/Å^2$, at the end of the simulation (40 ns) the protein is still attached to the surface. The dissipated energy will be higher than the value reported here for this spring constant. As a result, in the case of a very flexible bond between the OC and the rest of the bone hierarchal structure the dissipation energy will be higher.

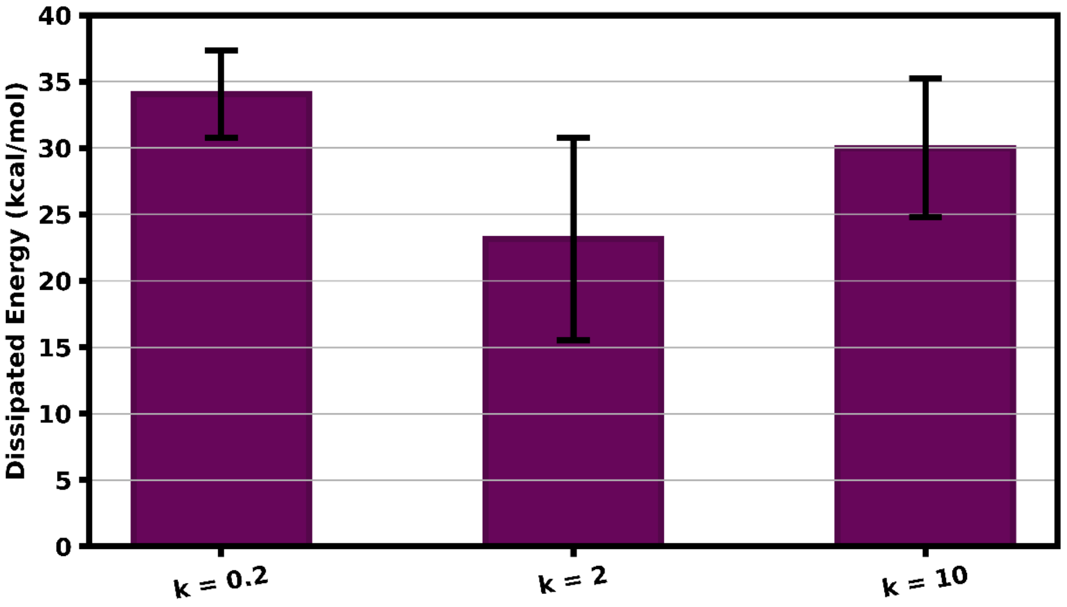

Figure SI4. The dissipated energy of the simulation R1-4A-#2 for parallel pulling with different SMD spring constants. The energy dissipation is the same for different spring constants

References

1. J. B. Thompson, H. G. Hansma, P. K. Hansma and K. W. Plaxco, *Journal of Molecular biology*, 2002, **322**, 645-652.
2. G. E. Fantner, J. Adams, P. Turner, P. J. Thurner, L. W. Fisher and P. K. Hansma, *Nano letters*, 2007, **7**, 2491-2498.
