# Supplementary figures and images for: The structural role of osteocalcin in bone biomechanics and its alteration in Type-2 Diabetes
Source: Sci Rep. 2020 Oct 14;10:17321. doi: 10.1038/s41598-020-73141-w (PMC7560881; doi:10.1038/s41598-020-73141-w)

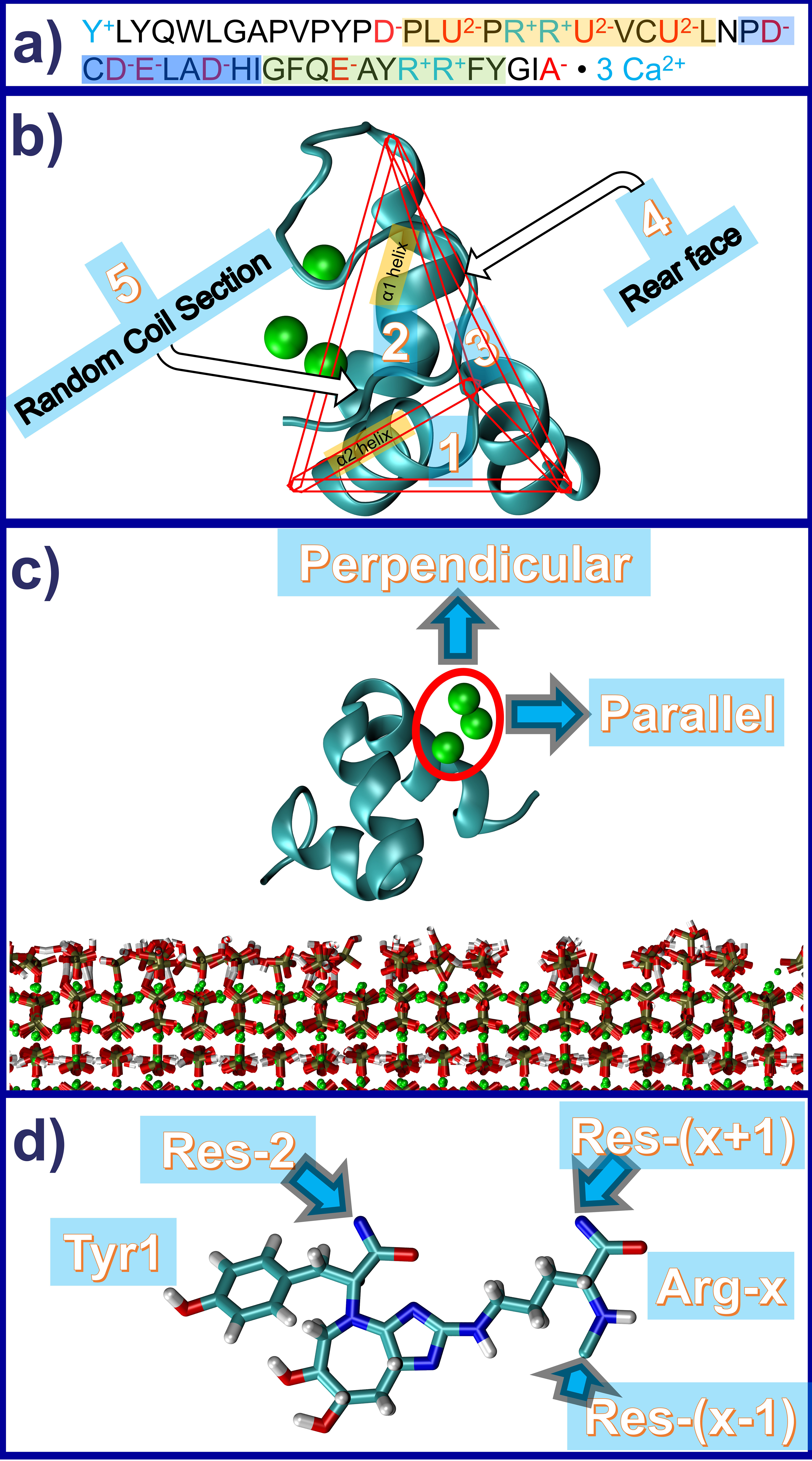

Supplement: Supplementary file 1 — Supplementary Information 1. [file 41598_2020_73141_MOESM1_ESM.zip › SI/Fig.1.tif]

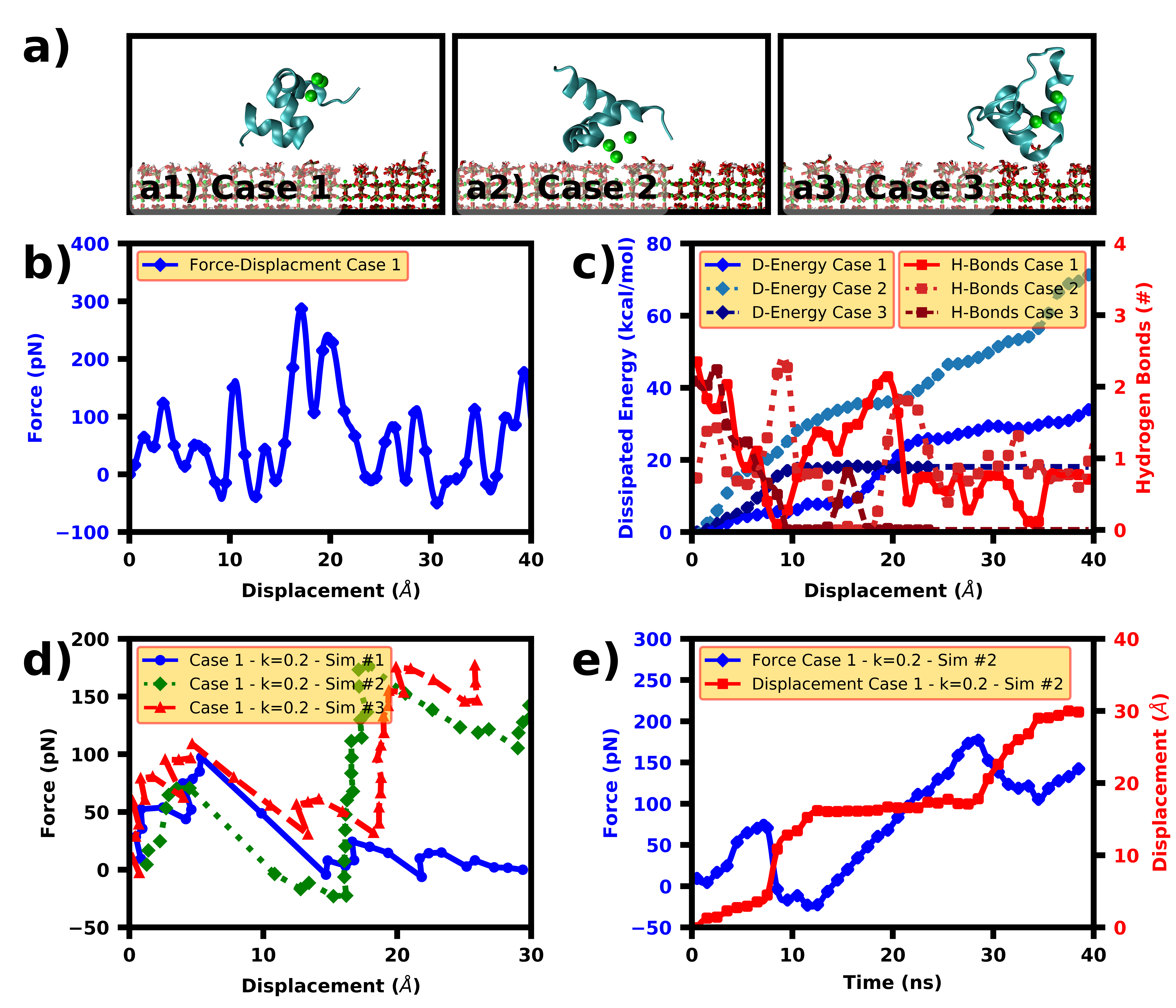

Supplement: Supplementary file 1 — Supplementary Information 1. [file 41598_2020_73141_MOESM1_ESM.zip › SI/Fig2_v02-Compressed.tif]

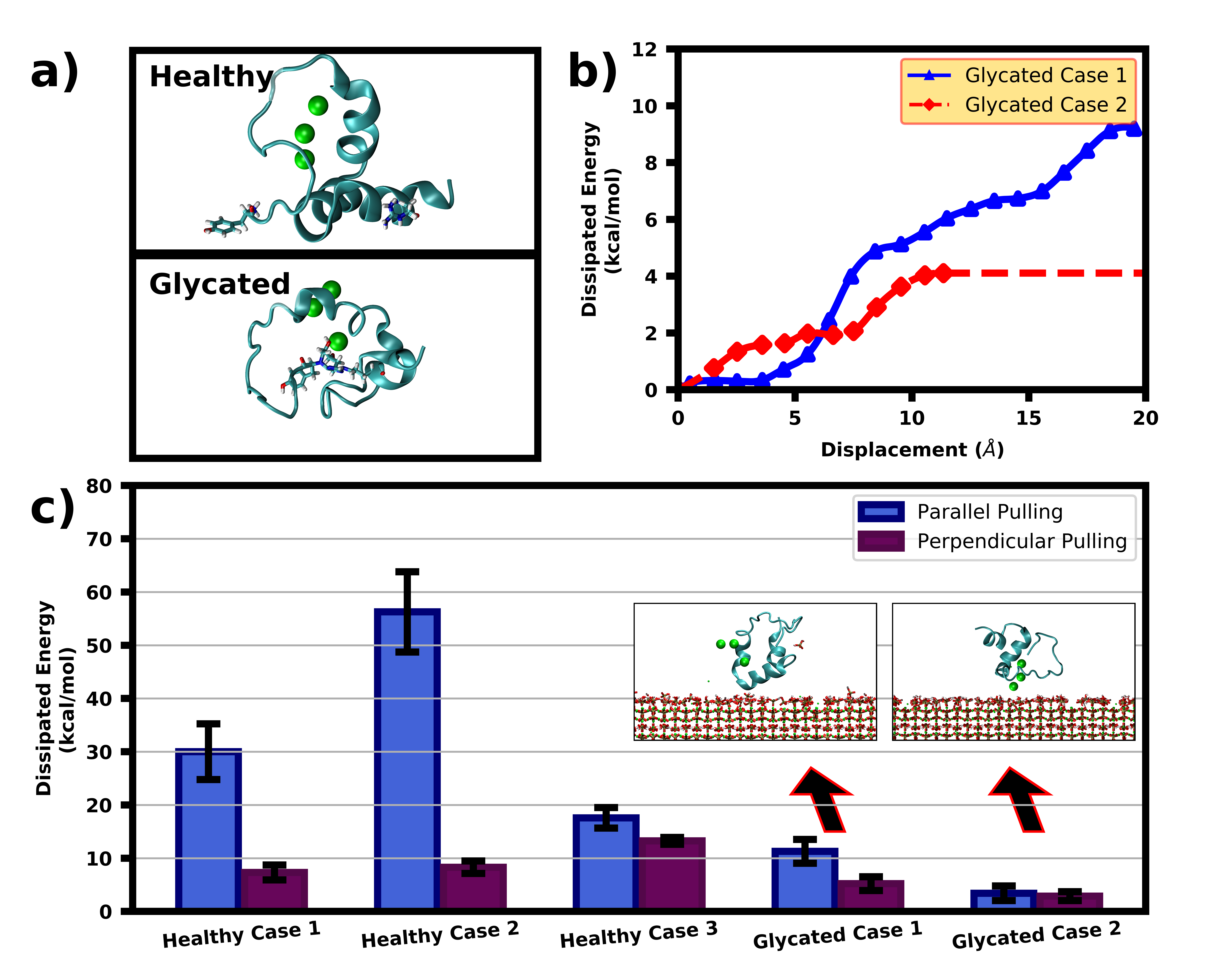

Supplement: Supplementary file 1 — Supplementary Information 1. [file 41598_2020_73141_MOESM1_ESM.zip › SI/Fig3_v2-Compressed.tif]

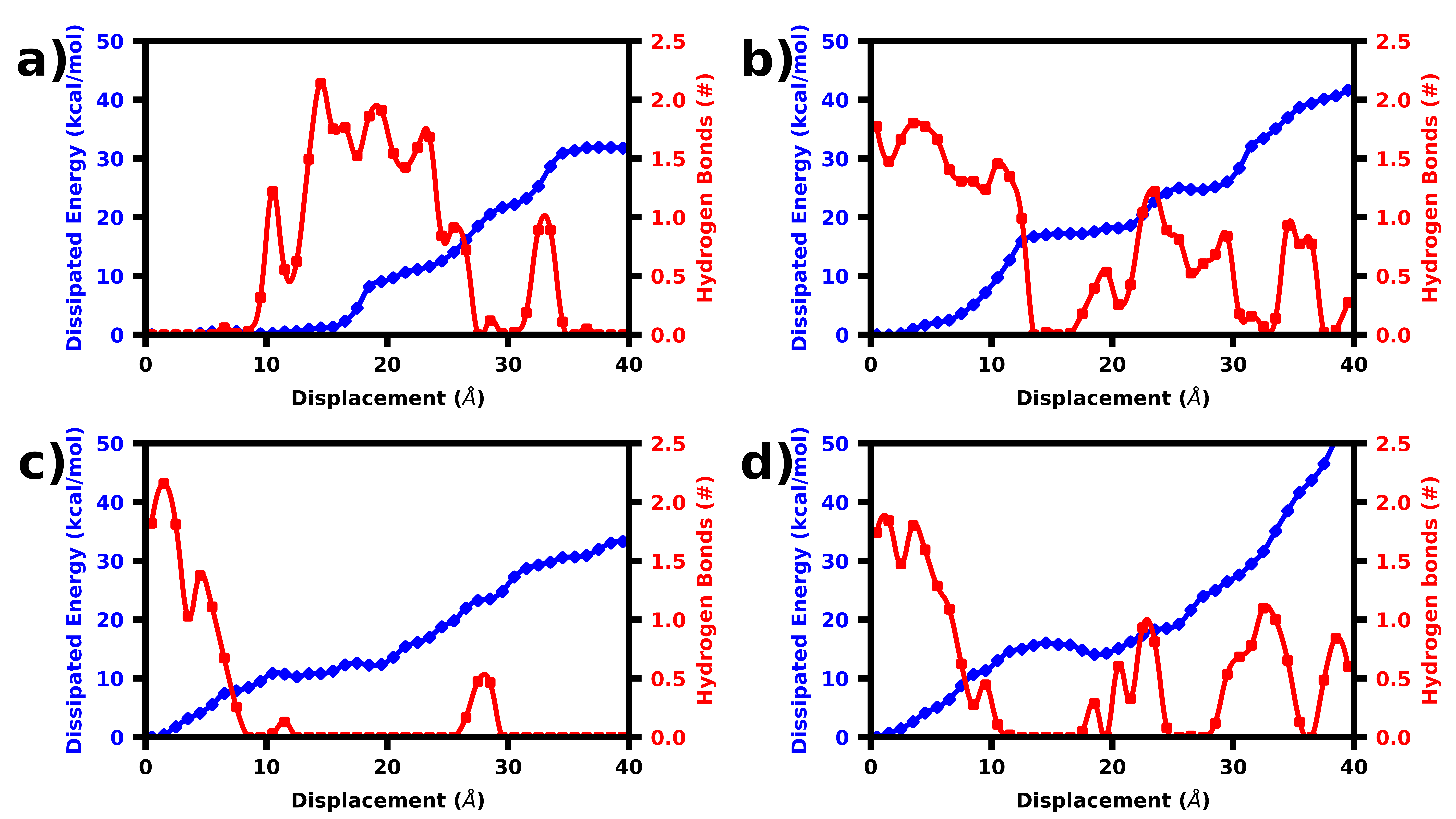

Supplement: Supplementary file 1 — Supplementary Information 1. [file 41598_2020_73141_MOESM1_ESM.zip › SI/FigSI1-Compressed.tif]

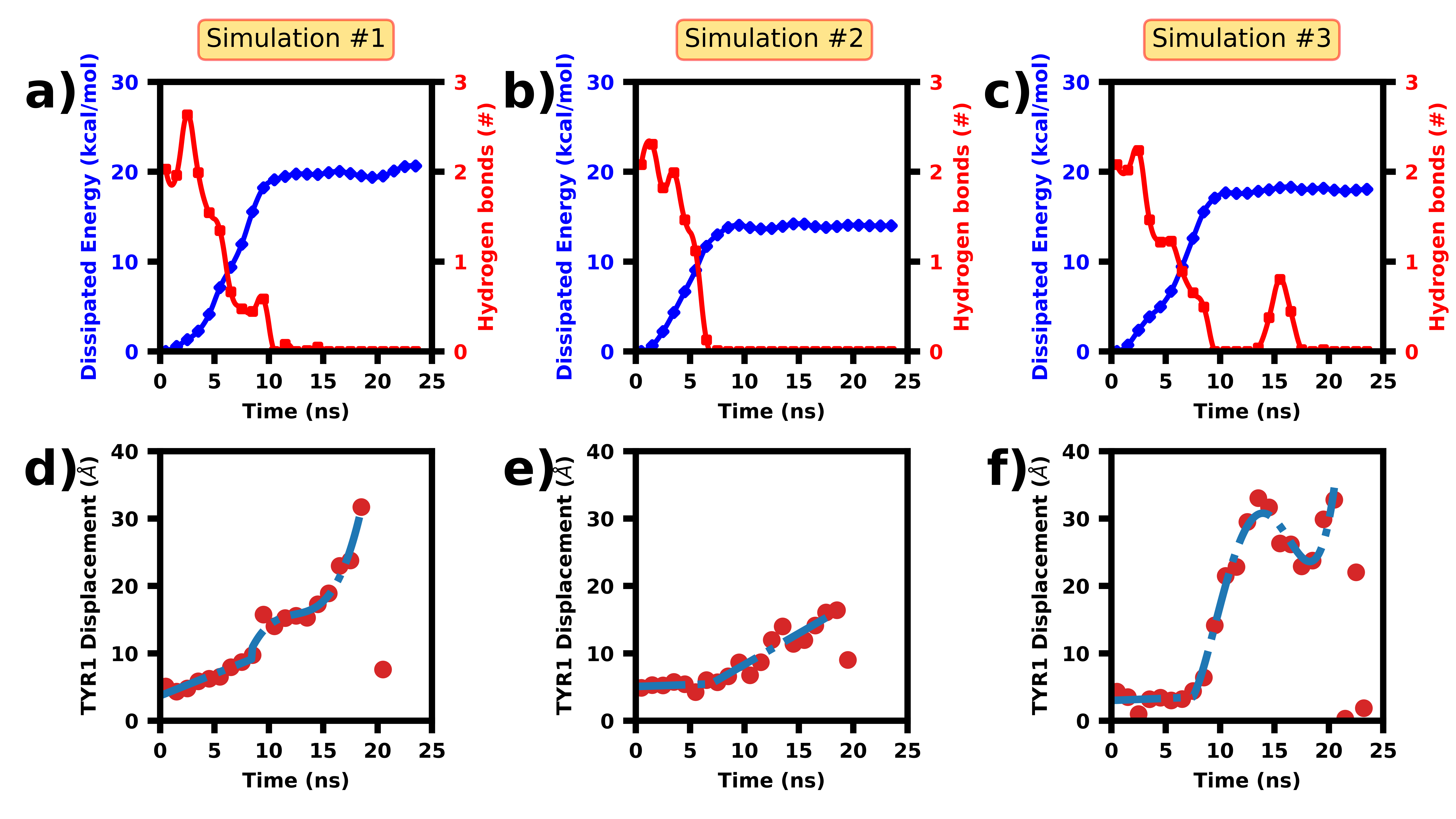

Supplement: Supplementary file 1 — Supplementary Information 1. [file 41598_2020_73141_MOESM1_ESM.zip › SI/FigSI2-Compressed.tif]

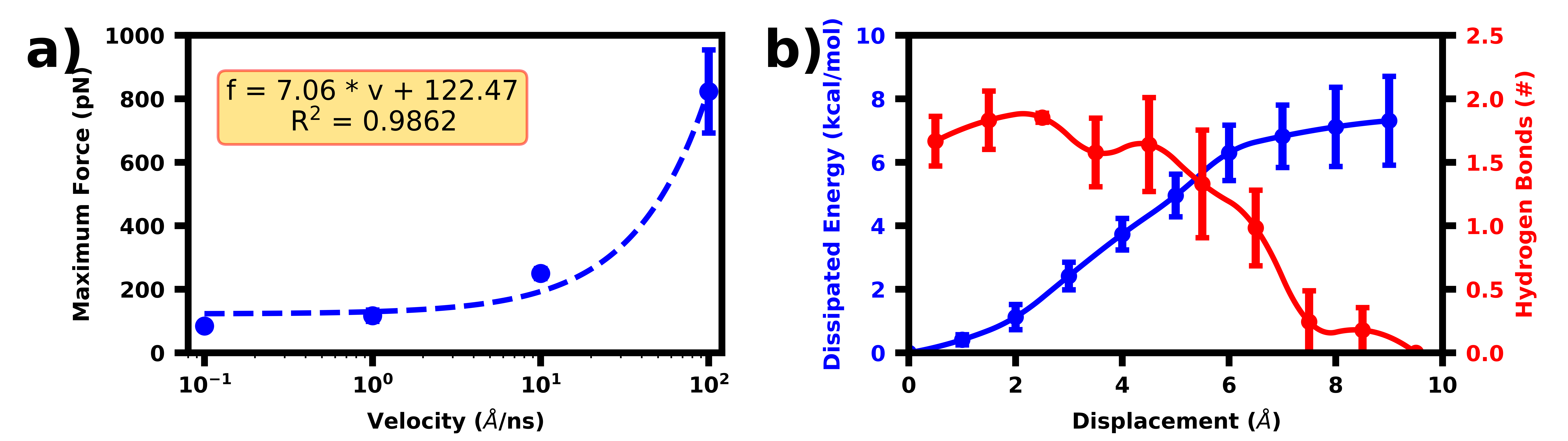

Supplement: Supplementary file 1 — Supplementary Information 1. [file 41598_2020_73141_MOESM1_ESM.zip › SI/FigSI3-Compressed.tif]

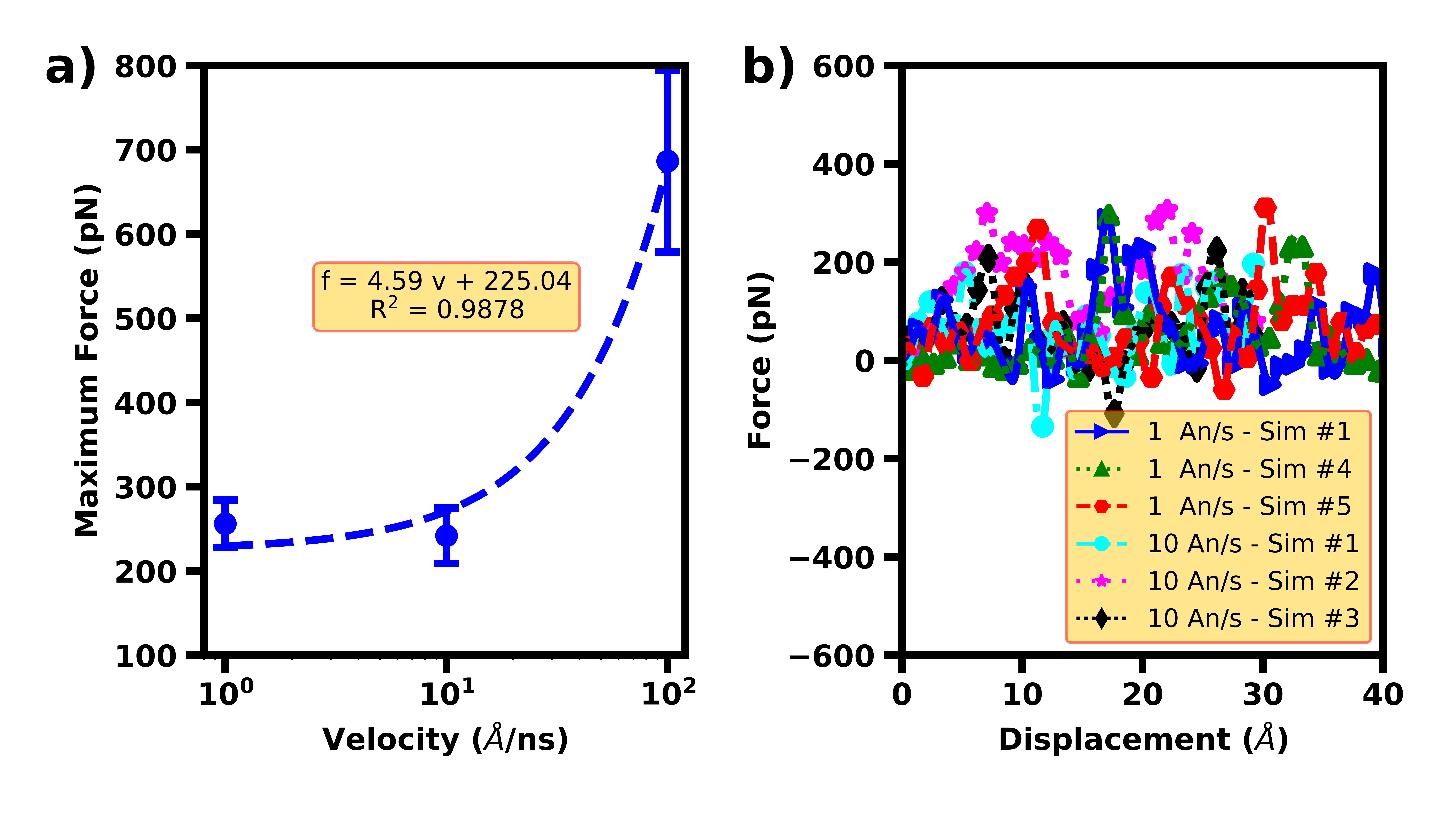

Supplement: Supplementary file 1 — Supplementary Information 1. [file 41598_2020_73141_MOESM1_ESM.zip › SI/FigSI4-Compressed.tif]

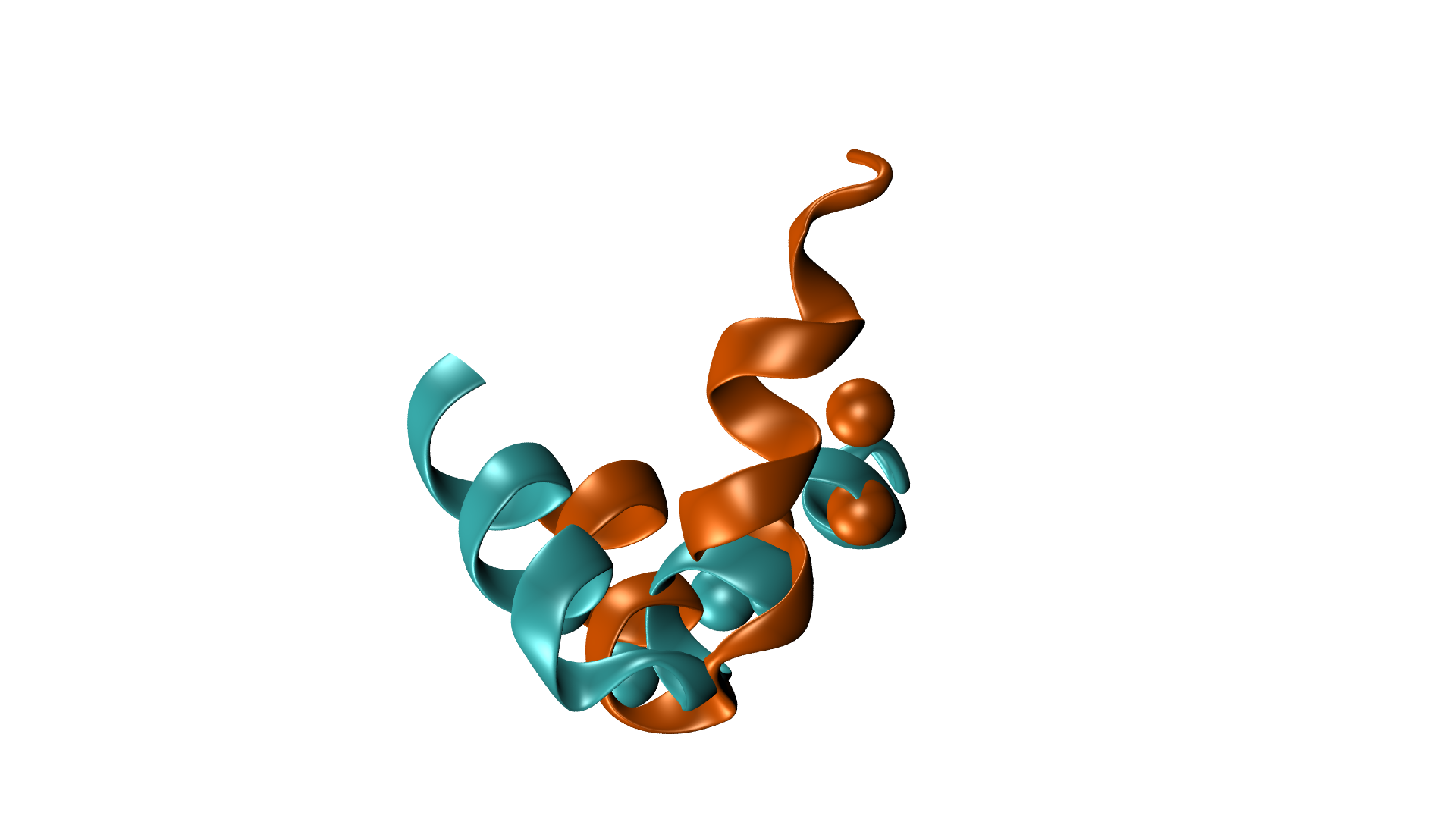

Supplement: Supplementary file 1 — Supplementary Information 1. [file 41598_2020_73141_MOESM1_ESM.zip › SI/FigSI5-Compressed.dat.tif]

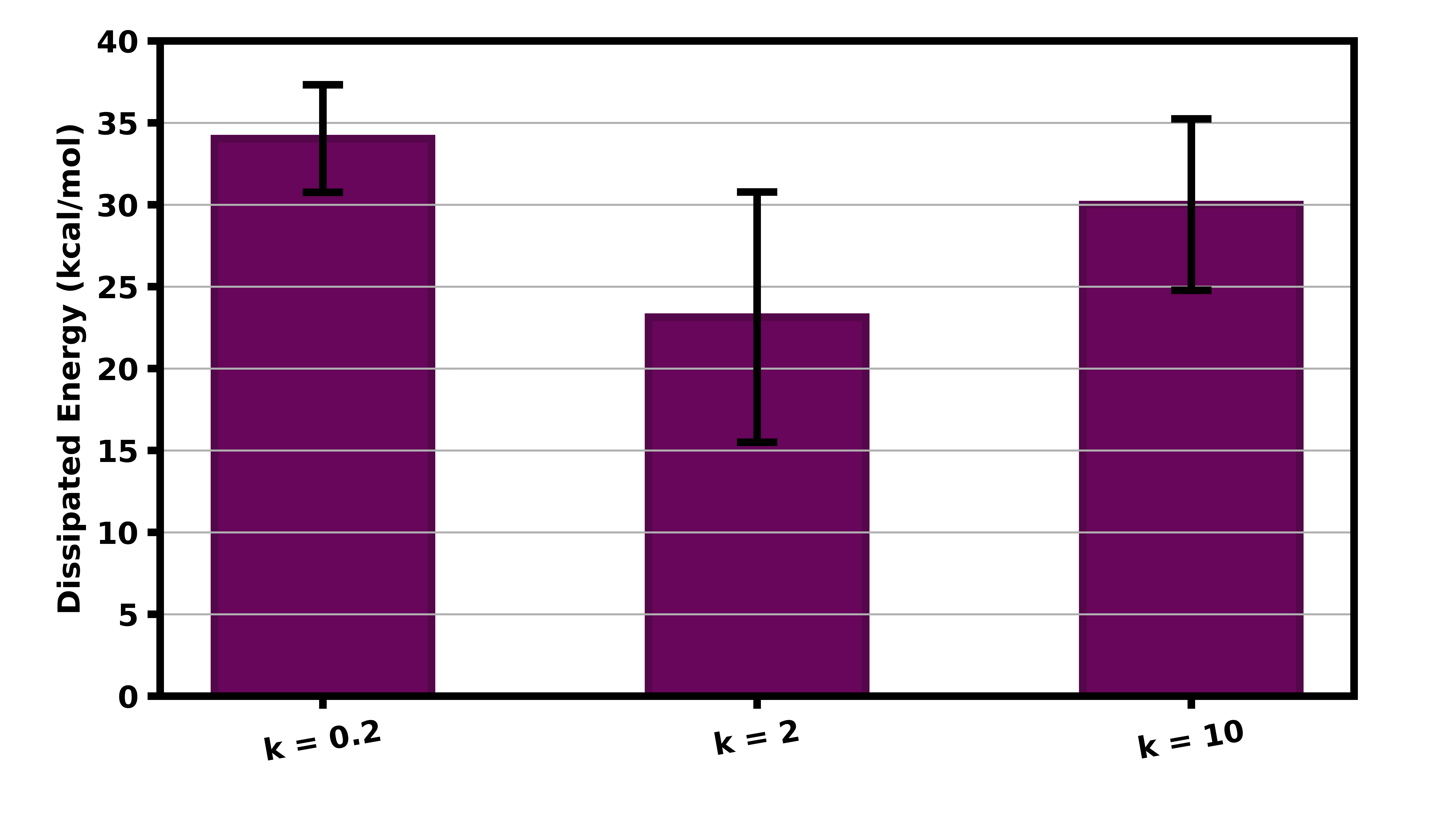

Supplement: Supplementary file 1 — Supplementary Information 1. [file 41598_2020_73141_MOESM1_ESM.zip › SI/FigSI6-Compressed.tif]

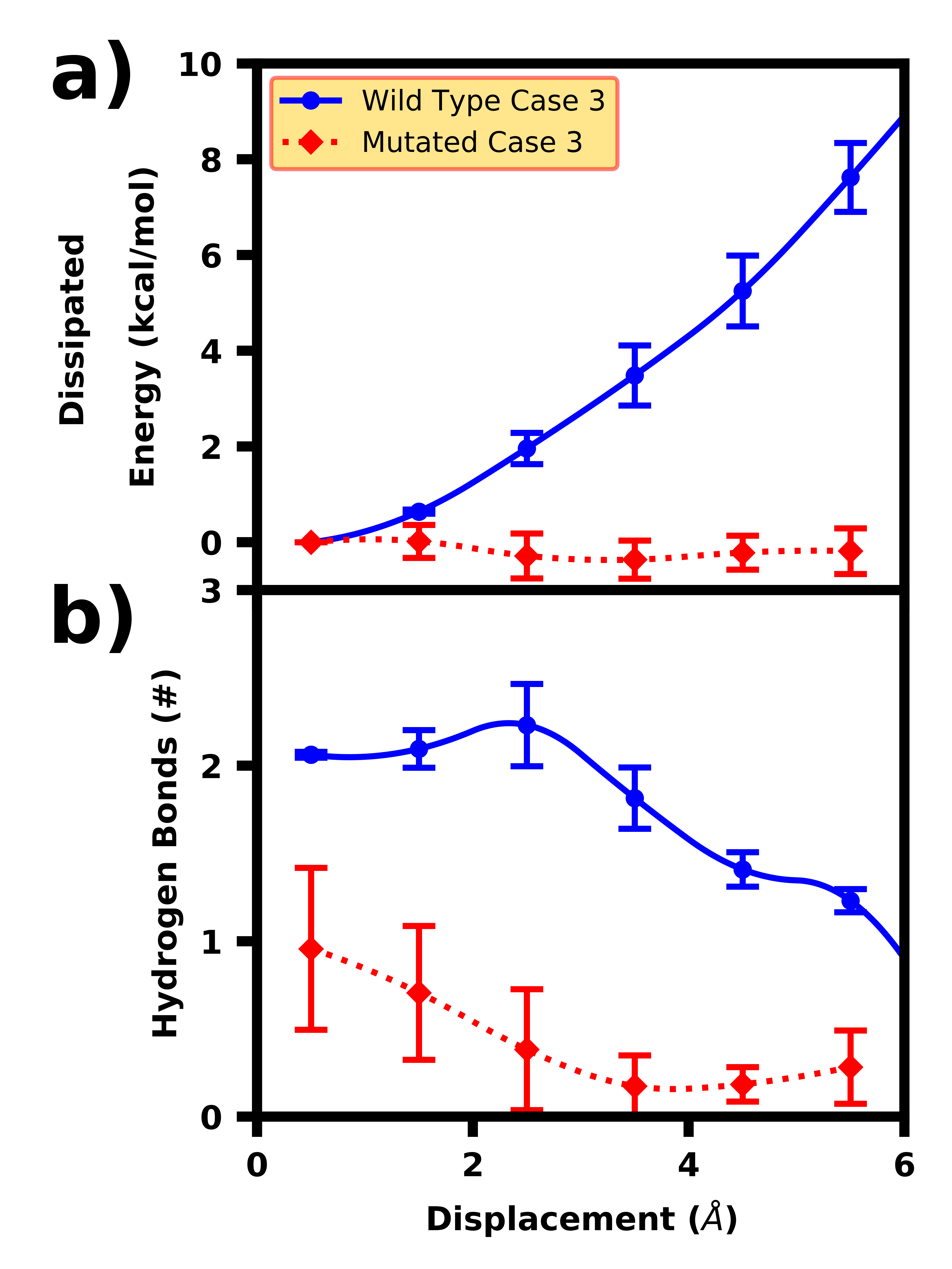

Supplement: Supplementary file 1 — Supplementary Information 1. [file 41598_2020_73141_MOESM1_ESM.zip › SI/FigSI7-Compressed.tif]
